# Effect of Ionic Liquids as the Mobile Phase Additives on the HPLC Resolution of Four Active Compounds from *Sophora flavescens Ait*

**DOI:** 10.3390/molecules14062127

**Published:** 2009-06-11

**Authors:** Minglei Tian, Junyu Liu, Kyung Ho Row

**Affiliations:** Department of Chemical Engineering, Inha University, Incheon, 402–751, Korea

**Keywords:** ionic liquid, retention, resolution, HPLC, *Sophora flavescens Ait*

## Abstract

The retention behaviour of four active compounds from *Sophora Flavescens Ait* using three ionic liquids as mobile phase modifiers was examined. The effect of the pH and the amount of ionic liquid modifier on the retention of these compounds was determined in methanol/water (v/v) as the mobile phase containing different ionic liquids ranging in concentration from 0.1 mmol/L to 3.0 mmol/L. The ionic liquids showed promise as additives in high-performance liquid chromatography.

## 1. Introduction

Ionic liquids (ILs) are organic salts that are liquids at ambient temperatures. Unlike traditional solvents, which can be described as molecular liquids, ionic liquids are composed of ions. Their unique properties such as non-volatility, non-flammability, and excellent chemical and thermal stability, make them an environmentally attractive alternative to conventional organic solvents [1,2,3,4,5]. Room temperature ionic liquids are gaining wide recognition as novel solvents in chemistry. Examination of their potential application in analytical chemistry, especially in separating analytes, is warranted because ILs offer some unique properties, such as negligible vapor pressure, good thermal stability, tunable viscosity, strong polarity, and miscibility with water and organic solvents, as well as good extractability for various organic compounds and metal ions [6].

Reversed phase HPLC is widely used as a standard analytical technque, and a number of stationary phases are commercially available [7]. In a chromatographic column, injected materials are separated based on their differences in the solvation and partition in a stationary phase [8]. HPLC is used for the separation, purification, collection of the single components, the qualitative and quantitative analysis of the adsorption, division, ion exchange, and exclusion effects when the mobile phase passes through the solid phase.

**Figure 1:**
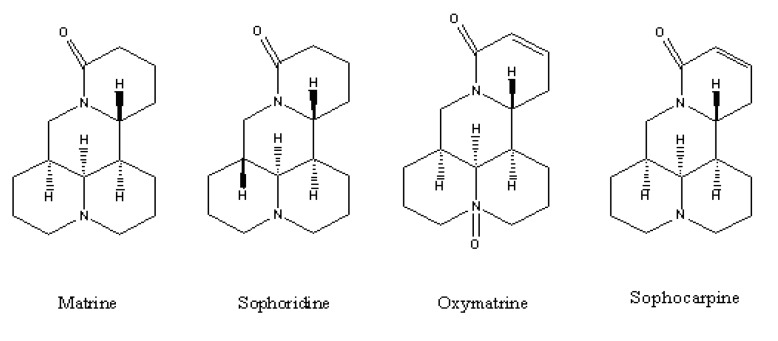
Chemical structure of the four compounds.

*Sophora flavescens Ait* (SFA) has been used since ancient times as a traditional Chinese herb to treat many diseases. It has recently attracted a great deal of attention in natural medication research on account of its high pharmacological activity. Sophora alkaloids, including matrine (MT), oxymatrine (OMT), sophocarpine (SC), sophoridine (SRI) and others [9] are its chief active components. Studies of its pharmacological effects have shown that SRI has wide range of pharmacological effects including anti-arrhythmic [10], anti-tumor [11], immunological enhancement [12], immunosuppressant [13], antiseptic and central nervous system excitation properties [14]. However, there are few reports showing that these four compounds can be separated at the same time, as it is difficult to separate these four alkaloids simultaneously due to their similar chemical structures (Figure 1). Therefore, a simple and effective analytical method for their simultaneous determination is needed.

This study examined the potential applications of different ionic liquids as additives in the separation of four compounds from SFA. Three types of ionic liquids were used as mobile phase additives in reversed-phase HPLC (RPHPLC) and the retention factors of the four compounds were determined using methanol/water as the mobile phase. The effects of the concentration of ionic liquids with their nature on the chromatographic retention and separation were examined.

## 2. Results and Discussion

In this study the retention factor was calculated using eq. (1):

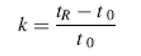
(1)
where t_0_ is the dead-time, t_R_ is the retention time, and k is the retention factor, respectively. 

The resolution (R) was calculated using the eq. (2):

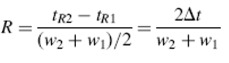
(2)
where t_R1_ and t_R2_ are the retention times of the first and second peak, respectively, and w_1_ and w_2_ are the corresponding peak widths.

### 2.1. Effect of ionic liquids types and concentrations on retention of four compounds

As reported by other authors [15], ionic liquid cations in the eluent will adsorb onto the C_18_ column packings and cause changes in their properties. Imidazolium cations can interact with silanol groups and compete with the polar group of analytes for the silanol groups on the alkylsilica surface in a column. Therefore, it can effectively shield residual silanols and improve the peak shapes, while also decreasing the retention time of the analytes. Another application of ionic liquid in chromatography is also for the suppression of deleterious effects of free silanols by using imidazolium tetrafluoroborate ionic liquid [16].

**Figure 2:**
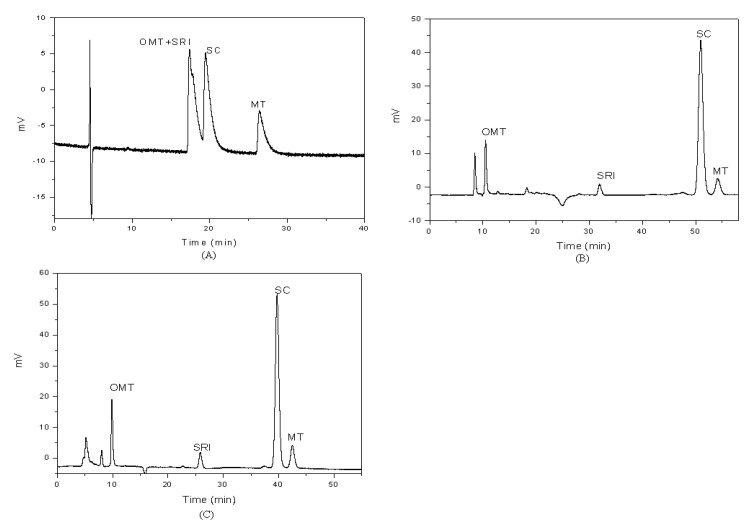
The chromatograms of standard compounds of OMT, SRI, SC and MT in methonal/water (45/55, v/v) as the mobile phase without modifier (A), with diethylamine (B) and with diethylamine and IL (C).

In this step, the influences of the three types of ionic liquids with different concentrations (0.1- 3.0 mmol/L) were tested. Figure 2 shows there chromatograms of mixture of four standard compounds with different mobile phases. In the beginning, the target compounds could not be separated well without a modifier (Figure 2A). With the addition of diethylamine and IL to the mobile phase, the resolution and peak shape were obviously improved (Figure 2 (B)) and the IL could decrease the retention times of the four compounds (Figure 2 (C)).

**Figure 3:**
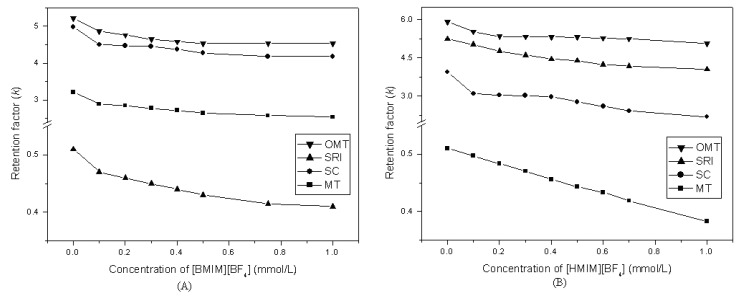
Effect of *k* of four compounds by using different concentrations of [BMIM][BF_4_] (A) and [HMIM][BF_4_] (B) as the mobile phase additives.

When the ILs were used, the four compounds can be separated except with [OMIM][BF_4_] as the mobile phase additive, so the pH of the mobile phase containing [OMIM][BF_4_] was modified in further experiments. Figure 3(A) and Figure 3(B) show the results with different concentrations of [HMIM][BF_4_] and [BMIM][BF_4_]. From these figures, we can see that the retention factor of the four compounds decreased as the concentration of ionic liquids increased. It appears that [HMIM][BF_4_] provides a better resolution with a decrease in alkyl chain length and the optimum concentration of the ionic liquid was 0.1 mmol/L.

### 2.2. Effect of pH of mobile phases with ionic liquids on retention of four compounds

**Figure 4:**
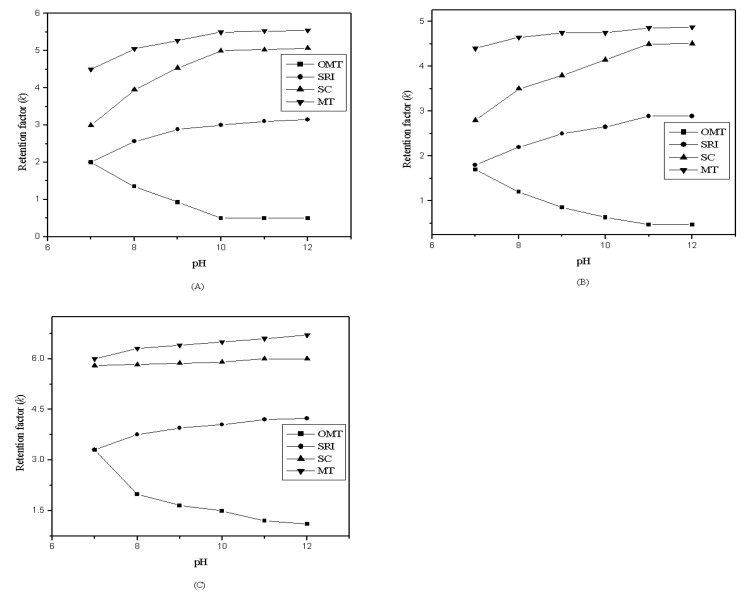
Effect of *k* of four compounds by using different pH of the mobile phase with 0.1 mM of [HMIM][BF_4_] (**A**), [BMIM][BF_4_] (**B**) and [OMIM][BF_4_] (**C**).

The pH increased slightly when the ionic liquids were added to the mobile phase because the tetrafluoroborate ([BF_4_]) is weak basic anion. However, the four compounds were alkaloids, so the mobile phase with a pH > 7.0 was more suitable for separating them. According to this condition, pH of the mobile phases with 0.1 mmol/L ionic liquids were examined (Figure 4). 

**Figure 5:**
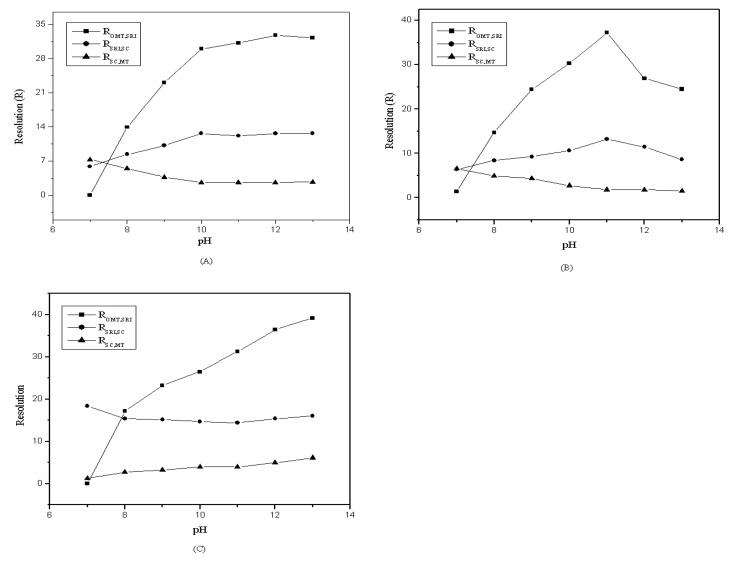
Effect of resolution of four compounds by using different pH of the mobile phase with 0.1 mmol/L of [HMIM][BF_4_] (**A**), [BMIM][BF_4_] (**B**) and [OMIM][BF_4_] (**C**).

Figure 5 shows the resolution between each two compounds. The retention factors of OMT, SRI and SC increased with the concentration of diethylamine increasing except MT. The MT can be eluted out earlier but can not be separated from other compounds with higher pH. The result revealed that pH=11.3 was the optimum condition for separation these four compounds.

**Figure 6:**
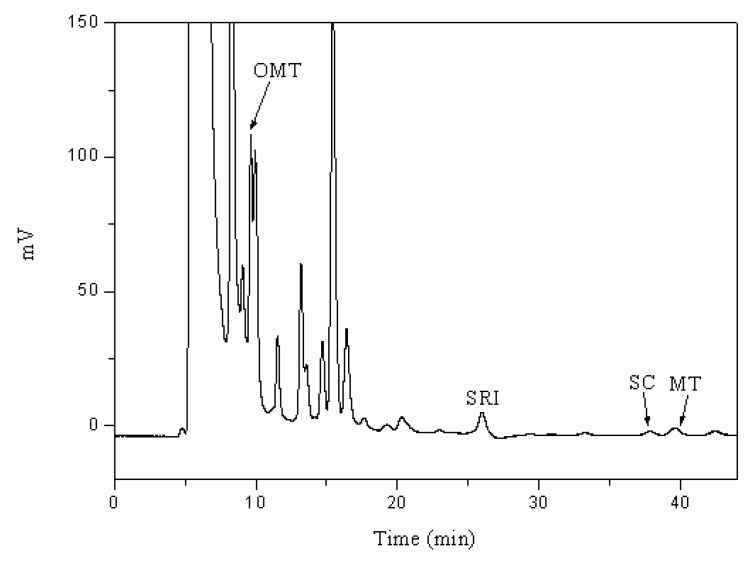
The chromatogram of four compounds in extract from SFA in methanol/water (45/55, v/v) as the mobile phase with 0.1 mM [HMIM][BF_4_] at the pH=11.3.

According to the results obtained in this experiment, the optimum mobile phase with additives was methanol/water (45/55, v/v) with 0.1 mM [HMIM][BF_4_] at pH=11.3. This condition was applied to separate the natural plant extract. Figure 6 shows that the four target compounds can be separated.

### 2.3. Method Validation

To ensure the specificity and selectivity of the condition, concentrations ranging from 5·10^-3^ to 1.0 mg/mL were applied for standards solutions of the four compounds. The analyte peak area of the analytes was plotted against the corresponding concentrations, and calibration curves were constructed using the least-squares method. The calibration curves of the four compounds showed good linearity (r^2^ > 0.998). The standard solutions were diluted and injected until the limit of detection (LOD) was obtained at a signal/noise ratio of 3, and 27 ng/mL, 35 ng/mL, 20 ng/mL and 32 ng/mL for OMT, SRI, SC and MT, respectively. Comparison with the real sample analysis verified that the values noted above were of acceptable precision and accuracy.

## 3. Experimental Section

### 3.1. Chemical regents

MT, OMT and SC were provided by the National Institute for The Control of Pharmaceutical and Biological Products of China, SRI was provided by Chengdu Mansite Biological Technology Co. Ltd., Sophora flavescens Ait was purchased from Anguo, Hebei, China (around August, 2008). Methanol, acetonitrile, *n*-propanol and diethylamine were all from Duksan Ltd., Korea. 1-Hexyl-3-methyl- imidazolium tetrafluoroborate ([HMIM][BF_4_]), 1-butyl-3-methylimidazolium tetrafluoroborate ([BMIM][BF_4_]) and 1-octyl-3-methylimidazolium tetrafluoroborate ([OMIM][BF_4_]) were purchased from C-TRI (Korea).

### 3.2. Apparatus

The chromatography system consisted of M930 Multi-solvent Delivery System, a variable wavelength M720 UV detector, the data processing was carried out with Autochrowin Ver. 1.42 (Young Lin Co. Korea) and a Rheodyne injector (20 μL sample loop). A C_18_ column (250 mm × 4.6 mm, 5 μm) was from RStech Corporation (Daejeon, Korea). Distilled water was filtered with a vacuum pump (Division of Millipore, Waters, U.S.A.) and filter (HA-0.45 μm, Division of Millipore, Waters, U.S.A.) before use. All the solvents should be filtered (MFS-25, 0.2 μm, WHATMAN, U.S.A.) before injection into the HPLC system. Mobile phase was methanol/water (45:55, v/v), the flow rate was 0.6 mL/min and UV wavelength was 238 nm.

### 3.3. Preparation of Standard solutions and extracts from SFA

Four standard compounds were mixed and dissolved in methanol to yield a final concentration of 0.25 mg/mL. The SFA had been dried at 30 °C, sliced and crushed into powder and 1.0 g was mixed with 25.0 mL water for 1 hr at 100 °C temperature. 

## 4. Conclusions

This paper discussed the effects of three ionic liquids as mobile phase additives for the HPLC separation of OMT, SRI, SC and MT from SFA. The length of the alkyl chain on the imidazolium ring has a significant inverse effect on the separation. Excellent separation condition of the four compounds was methanol/water (45:55, v/v) with 0.1 mmol/L [HMIM][BF_4_] as the additive at pH=11.3. The role of the ionic liquids is multiple and further research will be needed to explain some of the observed phenomena. In any case, ionic liquids show promising performance as additives in HPLC.
